# Modulation of Aire regulates the expression of tissue-restricted antigens

**DOI:** 10.1016/j.molimm.2007.05.014

**Published:** 2008-01

**Authors:** Vivian Kont, Martti Laan, Kai Kisand, Andres Merits, Hamish S. Scott, Pärt Peterson

**Affiliations:** a Molecular Pathology, Biomedicum, Tartu University, Ravila 19, 50411 Tartu, Estonia; b Institute of Technology, Tartu University, Tartu, Estonia; c Division of Molecular Medicine, The Walter and Eliza Hall Institute of Medical Research, Melbourne, Australia

**Keywords:** Autoimmune regulator, Thymus, Tissue-restricted antigens, Autoimmunity

## Abstract

Intrathymic expression of tissue-restricted antigens (TRAs) has been viewed as the key element in the induction of central tolerance and recently, a central role for the autoimmune regulator (*Aire*) has been suggested in this process. The aim of this study was to establish whether down or up-regulation of Aire leads to alterations in TRA expression and whether this is limited to thymic epithelial cells. This study also characterized whether TRAs follow Aire expression during normal development, and whether thymic microenvironment plays a role in the expression of Aire and TRAs. We did several in vivo and in vitro experiments to manipulate Aire expression and measured expression of four TRAs (Trefoil factor-3, Insulin-2, Major urinary protein-1 and Salivary protein-1) by real-time RT-PCR. Aire had an allele dose-dependent effect on TRA expression in the thymuses of mice from two strains, C57BL/6J and Balb/c, but had no effect on TRA expression in the lymph nodes. In the thymus, Aire and TRAs were both localized in the medulla and were co-expressed during normal development and involution. In the primary stromal cells as well as thymic epithelial cell line, the adenoviral over-expression of Aire resulted in an increase in TRA expression. By manipulating in vitro organ-cultures we showed that thymic microenvironment plays a dominant role in Aire expression whereas TRAs follow the same pattern. The data underline a direct role for Aire in TRA expression and suggest that modulation of Aire has a potential to control central tolerance and autoimmunity.

## 1 Introduction

Thymus has an essential role in establishing immune tolerance. Previous studies have demonstrated that tissue-restricted antigens (TRAs) are expressed in thymus and that this expression is needed for the deletion of self-reactive T cells (Kyewski and Klein, 2006). A central feature in this process is the promiscuous expression of TRAs by epithelial cells in thymic medullary region, where the TRAs are presented and encountered by the thymocytes, leading to the induction of tolerance either by clonal deletion or functional inactivation (Derbinski et al., 2001). In this context, medullary thymic epithelial cells (mTEC) highly express MHC molecules with costimulatory signals and act as professional antigen presenting cells (APCs) in thymus. A detailed study of the gene expression pattern in mTEC revealed that many of the TRAs and, in particular, almost all putative autoantigen targets of experimental animal models and human diseases are expressed by mTEC (Derbinski et al., 2001; Gotter and Kyewski, 2004). Altogether, the pool of promiscuously expressed genes in thymus appears to be highly diverse including tissue and sex-specific genes and genes specifically involved in development (Kyewski and Klein, 2006).

An important molecule in regulation of TRA expression in mTEC is autoimmune regulator (Aire) (Nagamine et al., 1997). The Aire protein has several features such as SAND and PHD finger domains that are characteristic to proteins involved in transcriptional control and has been reported to bind directly to DNA (Kumar et al., 2001) and to a common transcriptional regulator and histone acetyltransferase, CREB binding protein (CBP) (Pitkanen et al., 2000). In the thymus and cell lines, the Aire protein is subcellularly located to the nuclear bodies (Bjorses et al., 1999; Heino et al., 1999), which have been associated with several functions, including modulation of chromatin structure, transcriptional control, DNA repair and antiviral response (Everett and Chelbi-Alix, 2007). Initial studies have shown the Aire protein to be predominantly expressed in mTECs and suggest it has a role in regulation of immune tolerance (Blechschmidt et al., 1999; Heino et al., 1999). In humans, mutations in AIRE cause autoimmune-polyendocrinopathy-candidiasis ectodermal dystrophy (APECED), a syndrome characterized by the presence of autoantibodies to multiple self antigens and lymphocytic infiltration of endocrine glands, leading to autoimmune endocrine disorders (Perheentupa, 2006; Peterson and Peltonen, 2005). In agreement with the human disease, the Aire deficient mice have autoantibodies and tissue infiltration, although the full development of autoimmune disease appears to depend on the genetic background of the mouse (Anderson et al., 2002; Kuroda et al., 2005; Ramsey et al., 2002). The Aire deficiency affects negative selection since there is a complete failure to delete the organ-specific thymocytes in this mouse model (Liston et al., 2003). More importantly, the microarray analysis of mTEC population shows a decreased or abolished expression of multiple tissue specific genes in the Aire deficient mouse suggesting thus that Aire plays a role in modulating TRAs in the mTEC (Anderson et al., 2002; Derbinski et al., 2005; Jiang et al., 2005).

This study aims to further clarify whether Aire can directly regulate the TRA expression by analyzing the expression of four antigens in several experimental settings where Aire's expression has been modulated. The study aims to establish whether there is a dose-dependent correlation between the number of Aire allele copies and TRA expression level in thymic epithelial cells, and whether TRAs are co-expressed with Aire during thymic development and involution. We also studied whether the over-expression of Aire as a sole factor is sufficient to induce TRA expression and whether thymic microenvironment plays a role in the expression of Aire and TRAs.

## 2 Material and methods

### 2.1 Mice and cell cultures

**Fig. 1:**
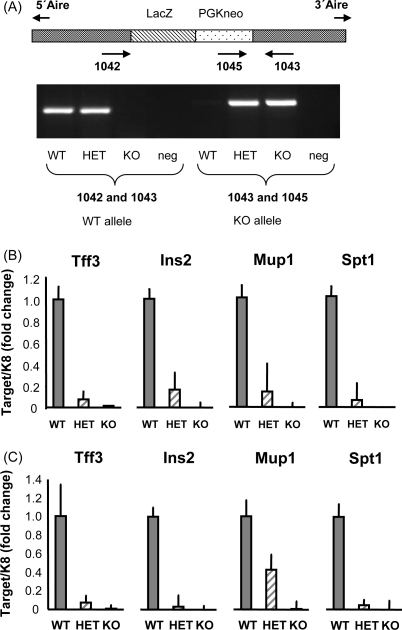
Dose-dependent effect of Aire on TRA expression in C57Bl/6 and Balb/c mice. Four to six weeks old C57Bl/6 or Balb/c mice were genotyped by PCR (A) and whole thymuses from WT, Aire HET and Aire KO mice were analyzed for TRA gene expression by real-time PCR. TRA expression followed the expression of Aire in a dose-dependent manner in C57Bl/6 (B) as well as Balb/c (C) mice. Data are mean with S.E.M. of triplicate measurements of one out of two representative experiments.

*Aire* deficient mice (C57BL/6J and Balb/c background) were generated at The Walter and Eliza Hall Institute (Melbourne, Australia). The inserted targeting construct containing LacZ gene replaced mouse Aire exon 8. For genotyping, the genomic DNA was extracted using JetQuick Tissue DNA Spin Kit (Genomed), and wild-type (WT) and knockout (KO) alleles were amplified using primers: 1042 5′-cagaagaacgaggat-3′, 1045 5′-cagactgccttggga-3′ or 1043 5′-ctgtcttctgtgaaggcttctagg-3′. As shown in Fig. 1A, primers pair 1042/1043 and 1043/1045 detect WT and KO alleles, respectively. Thymuses from 4- to 6-week-old WT, Aire HET (heterozygote) and Aire KO mice were used. Embryonic (E13.5, E15.5 and E17.5), newborn, neonatal D11 and adult (6 weeks, 6 months and 12 months) mouse tissues were used in developmental dynamics analysis. Mice were maintained at the mouse facility of the Institute of Molecular and Cell Biology, Tartu University. TEC 1C6 cell line (Mizuochi et al., 1992) was kindly provided by G. Holländer (University of Basel, Switzerland). Human embryonic kidney (HEK293) cells were cultured in Dulbecco's modified Eagle's medium (DMEM) supplemented with 10% fetal calf serum (FCS), 100 U/ml penicillin, 100 μg/ml streptomycin and 0.25 μg/ml amphotericin B (Gibco BRL).

### 2.2 EGFP and Aire adenovirus construction and infection

The pAdTrack-CMV (Stratagene) vector expressing enhanced green fluorescence protein (EGFP) gene was used as pAd-GFP plasmid. The mouse *Aire* gene was amplified from pcAire vector (Heino et al., 2000) using the primers: mAire-5-SalI 5′-tttgtcgac agatggcaggtggggatggaatg-3′ and mAire-3-NotI_stop 5′-tttgcggccgctcaggaagagaagggtggtgtc-3′ and cloned into SalI and NotI sites of pAdTrack-CMV resulting in AdAire-GFP. HEK293 cells (Invitrogen), which constitutively express AdEasy deleted E1 genes *in-trans*, were used for expression analysis of adenoviral vectors and for virus growth. To make recombinant adenoviruses, pAd-GFP and pAdAire-GFP plasmids were recombined with pAdEasy-1. The amplification and harvest process was repeated to generate higher titer viral stocks. Subsequently, the lyzates from several amplification steps were purified by CsCl gradient centrifugation (He et al., 1998). Virus bands was collected and mixed with 2× preservation buffer (10 mM Tris pH 8.0, 100 mM NaCl, 0.1% BSA, 50% glycerol). We quantified the Ad-GFP and AdAire-GFP virus particles by absorbance measurement at 260 nm to be equivalent to 10^12^ particles with virus titers >2 × 10^8^ pfu/ml. The adenoviruses were next verified for their expression by infection of HEK293 cells for 48 h and analyzed by immunoblotting with rabbit polyclonal anti-mouseAire and mouse monoclonal anti-G3PDH (Ambion). The signal was detected by Supersignal^®^ West Pico Chemoluminescent Substrate (Pierce Biotechnology) according to the manufacturer's instructions. For adenoviral infection of thymic primary culture and TEC 1C6, the cells were infected with Ad-GFP and AdAire-GFP at approximately 70% confluence. Cells were incubated in 500 μl serum-free OptiMEM (100 U/ml penicillin, 100 μg/ml streptomycin and 0.25 μg/ml amphotericin B) and infected with equal amounts of virus for 1 h. The infection rate was further assessed by quantitative real-time PCR analyzing intake of viral genomic DNA.

### 2.3 Thymic stromal cell isolation

Small cuts were made into the capsules of thymi dissected from eight to twelve 4-week-old mice and thymocytes were released by repetitive pipeting. The remaining thymic fragments were incubated in 0.5 mg/ml dispase/collagenase (Roche) and 1.5 μg/ml DNase I (AppliChem) in PBS at 37 °C for 20 min, with gentle agitation using a Pasteur pipette every 5 min. Released cells were collected to separate fractions and fresh enzyme solution was added four times. The cells were resuspended in 5 mM EDTA in 10 ml of RPMI-1460. Isolated cells were used either for stromal cell isolation or for growing thymic primary culture. For thymic primary culture, the isolated cells were cultured for 4 days in DMEM supplemented with 10% FCS. For stromal cell isolation cells were counted in each fraction. To obtain 2 × 10^8^ cells required for isolation, the final fraction was used first and then collected backwards through the collected fractions. The fractions were pooled and passed through a 100 μm mesh to remove clumps.

### 2.4 Cell sorting

For CD45 depletion CD45 MicroBeads (Miltenyi Biotec) were used according to manufacturers instructions. For cortical epithelial cell (cTEC) isolation, the CD45^−^ cells were stained with H213-HB Ab (anti-CDR1) followed by goat anti-rat IgG Microbeads (Miltenyi Biotech) and AutoMACS separation (isolation mode: Possel-S). The positive fraction (CDR1^+^) contained magnetically bound cTEC-s. For mTEC isolation CDR1^−^ cells were stained with G8.8 (anti-EpCAM, generated from a G8.8 hybridoma cell line) followed by goat anti-rat IgG Microbeads (Miltenyi Biotec) and separation as previously described. The purity of mTEC and cTEC was >80% as assessed by staining with anti-CD45 FITC (30F11, Miltenyi Biotech) and anti-I-A^b^ PE (AF6-120.1, BD Biosciences) using FACSCalibur flow cytometer (BD Biosciences).

### 2.5 Immunofluorescence and microscopy

Cryostat sections (5μm) of fresh-frozen 10-day-old mouse thymus were thaw-mounted onto SuperFrost Plus microscope slides (Menzel–Gläzer) and fixed in cold acetone (−20 °C) for 5 min. Sections were permeabilized in PBS/0.5% Triton X-100/1% normal goat serum (DAKO) for 15 min. Slides were blocked with 1% normal goat sera for 20 min at room temperature and incubated with rat monoclonal G8.8 (1:100) or rabbit polyclonal anti-mAIRE (1:2000) and then incubated with Alexa Fluor 594-conjugated goat anti-rat IgG (H + L) or Alexa Fluor 488-conjugated goat anti-rabbit (Fab)_2_ (both from Molecular Probes, Eugene), followed by washing six times in PBS. The slides were incubated with 15 μg/ml DAPI (Roche) and mounted with fluorescent mounting medium (DAKO). The images were acquired by fluorescence microscopy (Eclipse TE2000-4; Nikon, Melville, NY).

### 2.6 Thymic reaggregate organ culture

Reaggregated thymic organ-cultures were done as described previously (Jenkinson et al., 1992). Briefly, thymic stromal cells from E17.5 C57BL/6 mice were prepared by disaggregating fetal thymic lobes, which had been previously cultured for 7 days in 1.45 mM deoxyguanosine (Sigma, St. Louis, MO) using 1× trypsin (Life Technologies, Grand Island, NY). Reaggregates were formed by mixing together stromal cells and thymocytes at a 3:1 cell ratio and cultured for 3 days at 37 °C.

### 2.7 Real-time PCR

RNA was isolated using TRIzol (Invitrogen, Life Technologies) and reverse-transcribed to cDNA using the SuperScript™ III Reverse Transcriptase (Invitrogen, Life Technologies). Real-time PCR was performed with the ABI Prism 7900HT Fast Real-Time PCR System instrument (Applied Biosystems) using qPCR SYBR Green Core Kit (Eurogentec) according to the manufacturer's instructions except that 2 mM MgCl_2_ concentration was used. The amplification program included an initial denaturation step at 95 °C for 10 min, followed by denaturation at 95 °C for 15 s, and annealing and extension at 60 °C for 1 min, for 45 cycles. SYBR Green fluorescence was measured after each extension step, and the specificity of amplification was evaluated by melting curve analysis. Primers used to amplify specific gene products from murine cDNA were K2-8 sense, 5′-aggagctcattccgtagctg-3′; K2-8 antisense, 5′-tctgggatgcagaacatgag-3′; Aire 11/12, 5′-ccccgccggggaccaatctc-3′; Aire 12/13, 5′-agtcgtcccctaccttggcaagc-3′; Tff3 sense, 5′-tacgttggcctgtctccaag-3′; Tff3 antisense, 5′-cagggcacatttgggatact-3′; Ins2 sense, 5′-gacccacaagtggcacaac-3′; Ins2 antisense, 5′-tctacaatgccacgcttctg-3’, Mup1 sense, 5′-tctgtgacgtatgatggattcaa-3’; Mup1 antisense, 5′-tctggttctcggccatagag-3′; Spt1 sense, 5′-aacttctggaactgctgattctg-3′; Spt1 antisense, 5′-gaggcctcattagcagtgttg-3′. The relative gene expression levels were calculated using the comparative *C*_t_ (ΔΔ*C*_t_) method (according to Applied Biosystems), where the relative expression is calculated as $2^{-\Delta \Delta C_{\text{t}}}$, and where *C*_t_ represents the threshold cycle. Every sample was run in three parallel reactions.

## 3 Results

### 3.1 Decrease in Aire expression down-regulates the TRA expression in a dose-dependent manner

We initially set out to confirm by real-time PCR the previously published array results (Derbinski et al., 2005) demonstrating decreased TRA mRNA levels in the *Aire* deficient mouse. In order to study whether the self antigen expression is dependent on *Aire* we chose four TRAs; *Tff3*, *Ins2*, *Mup1* and *Spt1*, which were downregulated in the Aire deficient mouse according to publicly available microarray data (Derbinski et al., 2005). Throughout the study, we normalized our data to the expression level of keratin 8 (K8) mRNA, which in thymus is specifically expressed in epithelial cell fraction and is not influenced by Aire gene expression (Anderson et al., 2002; Derbinski et al., 2005). The real-time PCR analysis showed almost complete absence of TRA mRNA signal in C57BL/6 Aire KO thymus samples, which was uniformly seen with all four antigens studied (Fig. 1B). Furthermore, *Aire* allele dose-dependency was observed, as heterozygous mouse thymus consistently showed lower expression levels compared to the WT thymus levels. The expression level of all four TRAs in heterozygous mice thymus was approximately 10–20% of the expression level in WT mice. In order to determine whether Aire's effect on TRA expression depends on the genetic background, we also measured expression levels of the four TRAs in Aire KO and Aire HET mice backcrossed to Balb/c WT mice (Fig. 1C). Again, we observed a clear allele dose-dependency for all TRAs studied and almost no expression of the TRAs in the Aire KO mouse.

**Fig. 2:**
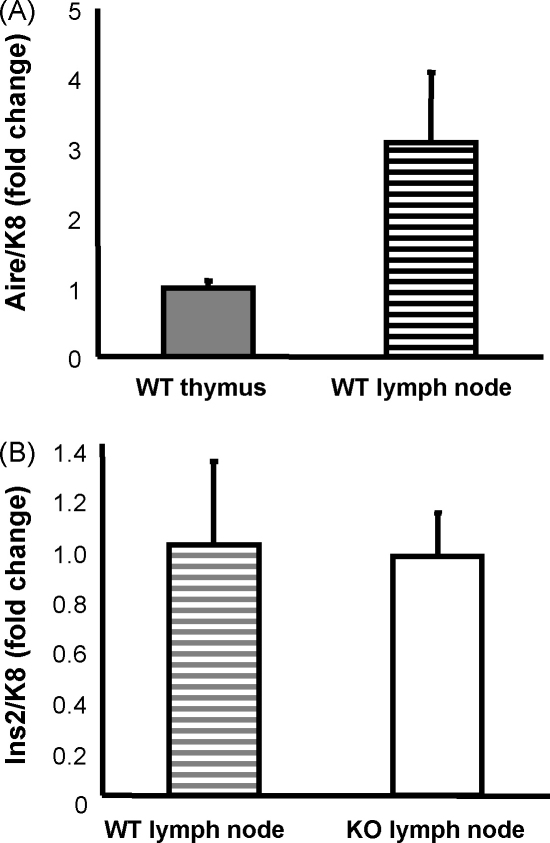
Expression of Ins2 in the lymph nodes of WT vs. Aire KO. Thymuses or inguinal lymph nodes were collected from 4- to 6-week-old mice and analyzed for Aire or Ins2 gene expression by real-time PCR. Aire expression relative to the epithelial marker, K8, was higher in the lymph nodes compared to the whole thymus of WT mice (A). The expression of Ins2 was unaffected in the Aire KO mice compared to the WT mice (B). Data are mean with S.E.M. of triplicate measurements of one out of two representative experiments.

We next determined whether Aire has a similar effect on TRA expression in the lymph nodes and quantified the expression of TRAs in the lymph nodes from C57BL/6 mice. Although we could clearly detect Aire mRNA in the lymph nodes at level that was even higher than the one of the whole thymus (Fig. 2A), most of the analyzed TRAs were undetectable or close to the detection limit. The higher expression of Aire in lymph nodes was relative to the epithelial cell marker K8, limiting the detection of Aire mRNA signal to the epithelial cell fraction. However, the mRNA signal for the Ins2 was clearly present in lymph node samples but, interestingly, did not depend on the presence of Aire (Fig. 2B).

**Fig. 3:**
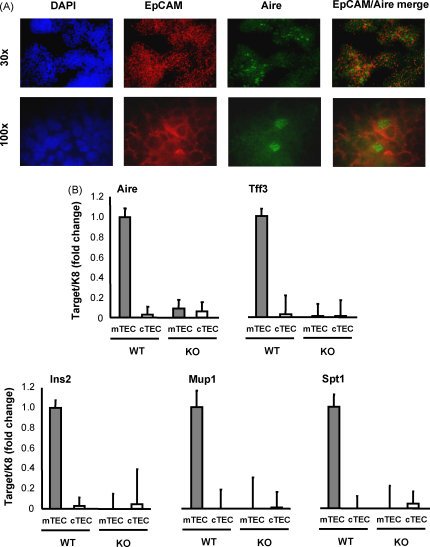
TRA expression in mTEC vs. cTEC populations of WT and Aire KO mice. Thymuses were stained with anti-EpCAM and anti-Aire antibodies and analyzed by immunofluorescent microscopy (A) or were enzyme digested and FACS-sorted according to the expression of EpCAM and analyzed for the expression level of TRAs by real-time PCR (B). Medullary compartment of thymus was distinctly characterized by high-EpCAM expression and by the presence of Aire-positive cells. TRAs were highly expressed in the thymic medulla but not in cortex of the WT mice. Aire KO mice showed virtually no expression of TRAs either in medulla or cortex. Data in (B) are mean with S.E.M. of triplicate measurements of one out of two representative experiments.

In order to establish whether Aire co-localizes in the thymus with TRAs, we purified the thymic mTEC based on the cell-surface marker EpCAM (Fig. 3A) and analyzed the expression of the TRA genes. As seen in Fig. 3B, the expression of the *Tff3*, *Ins2*, *Mup1* and *Spt1* antigens was limited to the mTEC population, i.e. the cell population of Aire expression. The cTEC population showed a very low expression for all four TRAs both in the WT as well as Aire KO mouse. Collectively these data show that Aire dose-dependently regulates TRA expression in thymus but not in the lymph nodes, and confirms by real-time PCR the previously published microarray data, suggesting that both Aire and TRAs are predominantly expressed in thymus medullary epithelium.

### 3.2 TRAs follow the expression of Aire during normal development and involution

**Fig. 4:**
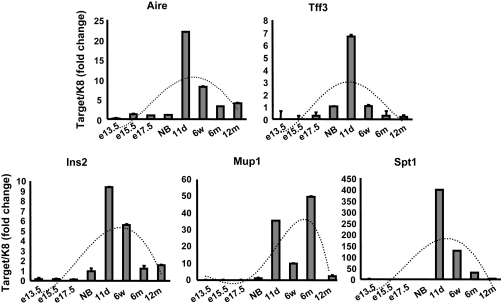
Aire and TRA expression during development. Thymuses were collected from normal WT mice at indicated time-points and the gene expression level analyzed by real-time PCR. Aire expression, as well as the expression of most of the ectopic genes reached their peak at D11 after birth followed by a gradual decrease. Data are mean with S.E.M. of triplicate measurements of one out of two representative experiments. Dotted line corresponds to polynomial estimation.

Thus far, the expression of Aire and TRAs has been studied in fetal or neonatal mice using WT versus Aire KO mice. If the expression of self antigens is directly dependent on Aire, this should be evident throughout the development of thymic tissue. However, the thymic cell content and volume changes significantly during development. To limit our analysis to the epithelial cell subsets only, we normalized our data again to the K8 gene. Thymuses from different embryonic, neonatal, young or adult developmental stages were analyzed for the Aire and TRA expression (Fig. 4). Very low Aire expression was detectable already at day E13.5 but showed a significant increase at E15.5. Thus, the start of Aire expression coincides with the influx of the first wave of the hematopoietic cells to the thymus. We observed the highest expression level at postnatal D11 and a gradual decrease thereafter until the very last time-point studied. Aire expression was, however, clearly present even in 12-month-old mice. The expression of TRAs closely followed the pattern of Aire reaching their peak at D11 followed by an obvious decrease, suggesting a role for Aire in their regulation.

### 3.3 Over-expression of Aire results in an increase in TRA expression

**Fig. 5:**
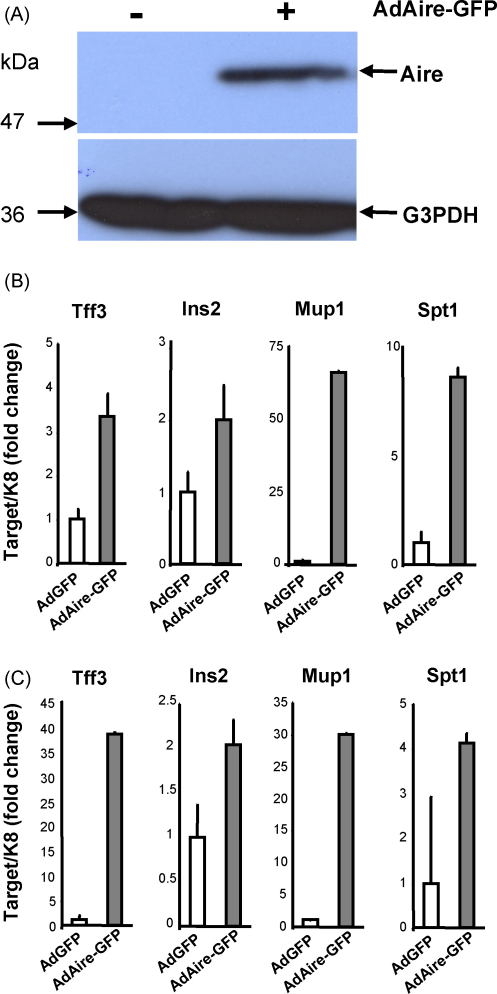
Over-expression of Aire induces TRA expression in primary thymic stromal cells and in thymic epithelial cell line. Infection with AdAire-GFP (as described in the methods) caused an induction of Aire protein (A). Monolayers of primary thymic stromal cells (B) or thymic epithelial cell-line TEC 1C6 (C) were infected with equal amounts of Ad-GFP or AdAire-GFP and, 48 h later, the cells were harvested for RNA purification and real-time PCR. Aire over-expression resulted in increased TRA expression in both cell types. Data are mean with S.E.M. of triplicate measurements of one out of two representative experiment.

Although the lack of Aire has been shown to have a negative effect on TRA expression, the ability of Aire as a single factor to up-regulate TRA expression has not been demonstrated. We used an adenoviral expression system (AdAire-GFP versus Ad-GFP) to determine whether the specific over-expression of Aire has any effect on TRAs. Infection with the AdAire-GFP resulted in an increased production of Aire protein (Fig. 5A). The AdAire-GFP infection also resulted in an increase of expression of all four TRAs studied, which was detected in primary thymic stromal cells (Fig. 5B) as well as in thymic medullary epithelial cell line TEC 1C6 (Fig. 5C). The results demonstrate that Aire can indeed act as a single inducer of TRAs in thymic epithelial cells even in the absence of signals from other cell-types normally present in thymus.

### 3.4 Thymic microenvironment is needed for the expression of Aire and TRAs

**Fig. 6:**
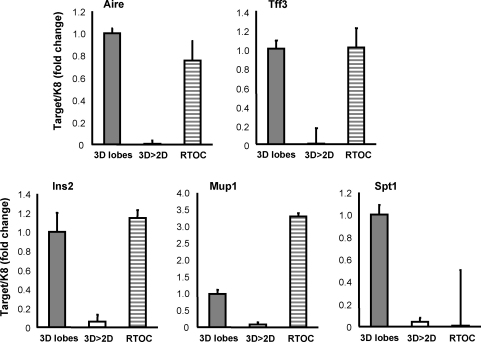
Requirement of thymic microenivironment for Aire and TRA expression. Thymuses from E17.5 old embryos of C57BL/6 mice were collected and either left intact (3D lobes), disaggregated (3D > 2D) or disaggregated and re-aggregated (RTOC) as described in the methods. Disaggregation resulted in down-regulation of Aire and TRA expression. This effect was restored by reaggregation. Data are mean with S.E.M. of triplicate measurements of one out of two representative experiment.

Although the precise mechanism is not fully understood, it has been demonstrated that the complex 3D structure of thymus is essential for mTECs to function properly (Anderson et al., 2006). To study whether the microenvironment plays a role in Aire and TRA expression, we used *ex vivo* culture of thymocyte-depleted dissaggregated and reaggregated thymic stromal cells. As shown in Fig. 6, the disaggregation of thymus to 2D culture resulted in a dramatic decrease in expression of Aire as well as most of the TRAs. This effect was, at least partly, reversed by reaggregation suggesting a critical role for the thymic microenvironment in Aire as well as TRA expression.

## 4 Discussion

In this paper, we report Aire dependent expression of four TRAs in mouse thymus. TRA expression was previously reported to be substantially decreased in Aire deficient mTEC subpopulations by microarray analysis (Anderson et al., 2002; Derbinski et al., 2005), which prompted us to follow the expression of four TRAs as marker antigens. All four genes have highly selective tissue specific expression. For example, Tff3 is restricted to mucin producing epithelial cells, with high expression in stomach and intestine (Hoffmann and Jagla, 2002; Karam et al., 2004), Spt1 is expressed in salivary and lacrimal glands (Dickinson et al., 1989) and Mup1 is expressed in liver but also in salivary, lacrimal and mammary glands (Shaw et al., 1983). Of the two insulin genes present in the mouse genome and encoded from separate loci, we selected Ins2 due to its high expression in the thymus as well as pancreatic beta cells, and previously reported Aire dependent expression pattern (Chentoufi and Polychronakos, 2002; Derbinski et al., 2005). Furthermore, it has been reported that Ins2 deficient mouse with low insulin expression in thymus has T cell reactivity to proinsulin (Chentoufi and Polychronakos, 2002).

The expression signal of all four TRAs tested was readily detectable in whole thymus and sorted mTEC samples by real-time PCR analysis. Interestingly, the TRA expression in heterozygous mouse thymus, both on C57Bl/6 and Balb/c background, was repeatedly only 10–20% and not 50% of the expression level seen in WT mouse, which would be expected when one of the Aire genomic alleles remains intact. This expression at lower levels than expected in the heterozygous mouse suggests Aire haploinsufficiency in regulation of target TRA genes. In haploinsufficiency of transcriptional regulators, only one intact gene copy is not sufficient for the functional activity of the regulated target gene product. The phenomenon has been explained by a stochastic expression model where diploid cells have a higher probability than haploid cells in maintaining the abundance of an expressed gene product above a low threshold level (Cook et al., 1998; Kaern et al., 2005). Further support to Aire haploinsufficiency comes from the report by Liston et al (Liston et al., 2004) demonstrating that the loss of one copy of the Aire allele in TCR-insHEL double transgenic mice caused severe functional defects in negative selection of autoreactive T cells and resulted in pancreatic cell insulitis, with diabetes incidence comparable to the Aire KO mouse. It should be noted, however, that the activation of target genes by a transcriptional factor in vivo can be dependent on the specific gene and the physiological context, such as in the case of cardiac transcription factor Nkx2–5 (Jay et al., 2005).

A recent study reported that lymph node stroma can also express Aire as well as a range of TRAs and contributes accordingly to tolerance induction (Lee et al., 2007). Here we report that, although Aire as well as Ins2 expression is clearly present in the lymph nodes of WT mice, the lack of Aire does not lead to a decrease in Ins2 expression in the Aire KO mice. The data suggests that, unlike in the thymus, other Aire-independent factors are likely to control Ins2 expression in the lymph nodes. Regarding the rest of the TRAs studied, we found very low, if any, expression by real-time PCR, which did not allow us to quantify the changes.

In order to determine whether the thymic expression of Aire and TRAs follow the same pattern during development, we monitored the expression throughout mouse development from E13.5 to 12 months. We detected an increase in Aire expression at day E15.5, which is in concordance with earlier reported results (Sousa Cardoso et al., 2006). The expression was at its highest at D11 and decreased thereafter, but was still present even in 12-month-old mouse tissue. The expression of TRAs followed a similar pattern to Aire expression, indicating a correlation between the amount of Aire and TRAs. The data demonstrate that the dynamics of Aire and TRA expression closely follow the dynamics of thymic function in general, being most active during the postnatal period and followed by a gradual decline in activity (Gray et al., 2006).

Aire dependent TRA expression is further illustrated by adenoviral experiments enforcing Aire expression in thymic epithelial cells. We show that over-expression of Aire as a single factor is sufficient to induce the expression of all four TRAs studied, providing evidence that modulation of Aire can directly lead to alterations in TRA levels and may thus also affect the maintenance of central tolerance. The finding that Aire, in addition to primary thymic stromal cells, can also modulate TRA levels in the thymic medullary epithelial cell line, suggests that there is no need for other cell types for the Aire-induced up-regulation of TRAs to occur.

The disruption of normal thymic architecture is known to affect the expression pattern and functionality of thymus, and it has been suggested that interactions between epithelial cells and thymocytes control the development of the thymic microenvironment and T cell development (Van Ewijk et al., 1994). Although the maturation of thymic epithelial stroma during the fetal period apparently occurs independently of thymocyte-derived signals (Jenkinson et al., 2005) and is mainly regulated by thymic mesenchyme (Jenkinson et al., 2003), thymocytes deliver signaling molecules, which are needed to maintain the normal adult thymic microenvironment. For example, lymphotoxin that signals through the lymphotoxin receptor and directs the alternative NfkappaB pathway, is needed for development of the thymic medullary compartment. Consequently, lymphotoxin receptor deficient mouse thymus had subnormal levels of Aire and TRAs (Boehm et al., 2003; Chin et al., 2003). Thymic medullary atrophy and lower expression of Aire and TRAs have been reported in mouse models deficient in several genes involved in the NFkappaB pathway, such as TRAF6, NIK, RelB or p52 suggesting an important role of this pathway in development of thymic medulla (Akiyama et al., 2005; Burkly et al., 1995; Kinoshita et al., 2006; Zhang et al., 2006). A recent study suggests that Aire deficiency may also cause changes in the organization and composition of the medullary epithelial compartment (Gillard et al., 2007). Thus, it is presently unknown whether the reduced levels of TRA expression seen in Aire KO mice are predominantly the result of changes in transcriptional activity or changes in thymic epithelial cell development. In this study, we show a sustained Aire and TRA expression in 2-deoxyguonosine treated FTOC, which rapidly disappeared after the disruption of the three-dimensional thymic meshwork into two-dimensional culture. Aire as well as TRA expression was regained in RTOC, however, the presence of thymocytes did not further augment this effect. These results show that Aire and TRA expression is dependent on the three-dimensional structure of epithelial microenvironment. This expression, however, seems to be independent of the presence of thymocytes being in line with previous data demonstrating the Aire expression signal in RAG deficient and CD3etg26 transgenic mice, in which T-cell development is blocked (Jenkinson et al., 2005; Zuklys et al., 2000).

In conclusion, we show that Aire has a dose-dependent effect on TRA expression in thymus but not in the lymph nodes. Both, Aire as well as TRAs localize in the thymic medulla and are co-expressed during normal development and involution. We also show that Aire can directly induce TRA expression in medullary epithelial cells although the thymic microenvironment plays a crucial role for the maximal expression to occur. Our data suggest a clear correlation between the expression of Aire and TRAs and indicate that approaches to stimulate Aire expression in thymic epithelium could be considered to modulate tolerance induction to peripheral antigens.
